# “Internet+” pharmacy in psychiatric hospital amid COVID-19 and post-pandemic period: analysis and development

**DOI:** 10.3389/fpsyt.2024.1434966

**Published:** 2024-12-20

**Authors:** Weiming Xie, Fei Wang, Yayun Qian, Linghe Qiu, Qin Zhou, Yuan Shen, Jianhong Wu

**Affiliations:** ^1^ Department of Pharmacy, Affiliated Mental Health Center of Jiangnan University, Wuxi, Jiangsu, China; ^2^ Department of Pharmacy, Wuxi Central Rehabilitation Hospital, Wuxi, Jiangsu, China; ^3^ Department of Pediatrics, Changzhou First People’s Hospital, Changzhou, Jiangsu, China

**Keywords:** psychiatric, COVID-19 pandemic, “Internet+” pharmacy, China, analysis and development

## Abstract

**Objective:**

This study aims to explore the differences in “Internet+” pharmacy prescriptions in psychiatric hospitals before and after the outbreak of the coronavirus disease 2019 (COVID-19) pandemic. It also seeks to examine changes in patient healthcare behaviors in the post-pandemic era and to identify the potential role of “Internet+” pharmacy in improving the current healthcare system.

**Methods:**

Prescriptions from the “Internet+” pharmacy at The Affiliated Mental Health Center of Jiangnan University, collected between December 1, 2021, and November 30, 2023, were analyzed. The period was divided into four stages based on the COVID-19 pandemic’s progression in China. Descriptive statistical analysis was conducted on various prescription-related factors, including patient information, prescription type, disease distribution, medication type, frequency of medication use, pharmacist review time, and instances of irrational medication use.

**Results:**

A total of 2914 prescriptions were collected. The male-to-female ratio (MFR) varied significantly across different stages of the epidemic. In the pre-pandemic II period, females represented the highest proportion (66.10%, MFR 0.51), and individuals aged 18-39 made up the majority (56.70%) across all stages. The proportion of psycho-counseling prescriptions was highest in the pre-pandemic II period (76.74%), while the total number of psycho-counseling prescriptions was greatest during the epidemic, with 798 cases. A total of 38 diseases were involved, with depression accounting for the largest proportion (38.98%) at each stage, followed by the highest usage of antidepressants (49.60%). A total of 85 types of medications were used, with quetiapine representing the highest proportion before the epidemic (16.56%, 10.92%), while escitalopram accounted for the highest proportion after the epidemic (10.98%). The majority of patients (70.87%) took medication once daily. 42.23% of pharmacist review times were ≤1 minute, and the mean review time was longest in the post-pandemic period (6175.1 seconds). During the pre-pandemic and epidemic periods, the most common pharmacist review time occurred between 12:00 and 17:59 (41.46%), while in the post-pandemic period, the most common review time was between 18:00 and 23:59 (36.70%). The initial rate of irrational prescriptions was 37.85%. After manual review by pharmacists, the irrational prescription rate of Internet prescriptions decreased to 1.13%.

**Conclusion:**

The development of “Internet+” pharmacy has effectively addressed the medical needs of the relevant population and played a crucial role in combating the COVID-19 pandemic. Future advancements should focus on optimizing the allocation of healthcare resources and expanding innovative pharmacy services to broaden the developmental pathways of the ‘Internet+’ pharmacy ecosystem.

## 1 Introduction

The coronavirus disease 2019 (COVID-19) pandemic has posed a major challenge to global public health security in this century. Since its onset in early 2020, the pandemic has spread worldwide, resulting in a total of 770 million confirmed cases and 6.98 million deaths (1). In response, the Chinese government has implemented a series of effective measures, including social distancing and traffic restrictions, to curb the spread of virus (2, 3). However, daily life, particularly medical treatment, has been significantly impacted.

A review of 177 longitudinal and repeated cross-sectional studies showed that the prevalence of certain mental health problems during the pandemic was higher than before (4). Although this situation may improve over time, the limitations of epidemic prevention and control contributed to increased strain on medical resources in the early stages of the pandemic. Furthermore, a large number of healthcare providers had to support regions with severe outbreaks, further exacerbating the strain on medical resources. Studies on receiver biases have reported that, in addition to challenges such as a lack of protective equipment and heavy workloads, medical staff in China faced growing pressure to provide in-person care (5, 6). Additionally, patients with mental illness were likely more affected by the pandemic due to difficulties in accessing treatment and paying for care (7).

To effectively implement epidemic prevention measures and meet the medical needs of patients, countries around the world have actively explored the use of “Internet+” hospitals. For patients with mental illness, Katsuhiko Hagi emphasizes that telepsychiatry has demonstrated symptom improvement effects comparable to those of face-to-face treatment (8). Additionally, some studies have shown that telemedicine helps curb the spread of COVID-19 and increases access to healthcare for patients (9).

In December 2020, the National Health Commission issued a notice on further promoting “Internet+” healthcare, aiming to encourage localities to expand Internet medical services and fully leverage the role of “Internet+” hospitals in primary healthcare (10). To meet the ongoing medication needs of existing patients with mental illnesses and address the medical needs of new patients, our hospital launched an “Internet+” hospital and began providing online pharmaceutical services in October 2021. Patients can receive remote consultations from doctors at home, and prescriptions are sent directly to them through certified third-party logistics channels. Additionally, patients can pay online and settle their medical insurance in real time, significantly reducing the risk of COVID-19 transmission.

According to incomplete statistics, by June 2021, approximately 1,600 hospitals in China had obtained business licenses for “Internet+” hospitals (11). By June 2023, the number of “Internet+” hospitals in China had surpassed 3,000 (12). However, with the resumption of normal medical services in the post-pandemic period, it remains unclear whether the rapid growth of “Internet+” medical care has altered patients’ medical habits or strained resources. Therefore, it is crucial to investigate the changes in Internet prescriptions in psychiatric hospitals after the pandemic. Additionally, few studies have used the timeline of the COVID-19 pandemic to compare Internet prescriptions in psychiatric hospitals. This study provides a detailed analysis of outpatient pharmacy practices in “Internet+” hospitals before and after the epidemic, evaluates the level of “Internet+” pharmacy services, and offers insights for the development of a new pharmaceutical ecosystem in the future.

## 2 Material and methods

### 2.1 Data source

The “Internet+” hospital allows patients to receive online psychiatric treatment or psycho-counseling from a doctor. After the consultation, the doctor issues a prescription, which is transmitted to the pharmacist workstation for preliminary review, pending the pharmacist’s final approval. If the pharmacist does not accept the prescription within 10 seconds, the system automatically performs a review and returns the result to the doctor. The doctor then confirms the prescription. The pharmacist will conduct the final review during their available time, prepare the medication for the patient, and hand it over to a certified third-party delivery service for distribution. All relevant information is integrated into the hospital information system (HIS).

### 2.2 Data collection

Prescriptions from December 1, 2021, to November 30, 2023, were retrospectively analyzed and divided into four periods based on the progression of the COVID-19 pandemic in China: pre-pandemic I (December 1, 2021 - May 31, 2022), pre-pandemic II (June 1, 2022 - November 30, 2022), during-pandemic (December 1, 2022 - May 31, 2023), and post-pandemic (June 1, 2023 - November 30, 2023). In December 2022, the Chinese government announced the relaxation of COVID-19 control measures, leading to a six-month period of outbreak and decline, defined as the during-pandemic period. The first six months of the preceding year were defined as pre-pandemic I, and the subsequent six months as pre-pandemic II. The six months following the during-pandemic period were defined as the post-pandemic period. Basic patient information, prescription types, disease distribution, medication types, medication frequency, pharmacist review times, and instances of irrational medication use were collected and subjected to statistical analysis.

### 2.3 System prescription review rules and pharmacist prescription review

#### 2.3.1 System prescription review

We utilized the prescription review system developed by Mulaorenkang. This system refines its database rules based on drug package inserts, clinical guidelines, and drug interaction data to automatically identify potential irrational medication use, generating preliminary review results. The types of irrational prescriptions and their corresponding criteria are as follows: 1) Off-label Indications (OLI): The use of medications for diseases or symptoms not listed in the drug’s approved labeling by regulatory authorities. 2) Medication Errors in Special Populations (MESP): Errors or inappropriate medication use in specific populations (e.g., children, older adults, pregnant women, or individuals with multiple comorbidities) due to physiological, pathological, or drug interaction factors. 3) Drug-Drug Interactions (DDI): Interactions between two or more drugs as identified by the database, potentially leading to enhanced or diminished therapeutic effects or adverse reactions, thereby affecting treatment outcomes. 4) Usage and Dosage Errors (UDE): Instances where patients or healthcare professionals deviate from the recommended usage or dosage, potentially resulting in insufficient efficacy or adverse reactions.

#### 2.3.2 Pharmacist prescription review

Based on the system prescription review, trained pharmacists authorized to dispense medications conduct a secondary review of all prescriptions. The definitions of review time and review time points are as follows: 1) Pharmacist review time: The duration from the issuance of a prescription by the doctor to the review of the prescription by the pharmacist. 2) Pharmacist review time point: The specific time point at which the pharmacist reviews the prescription.

### 2.4 Statistical analysis

Descriptive statistics was used to evaluate the prescriptions. Excel 2016 was used for data entry and SPSS 22.0 was used for data analysis. Number of prescriptions, percentage, median (interquartile range, IQR), and mean were used for data description.

### 2.5 Bias control

All data were entered by two trained professionals to minimize information bias caused by human error or inconsistent entries. Additionally, data reliability was ensured through dual data validation and regular quality checks.

## 3 Results

### 3.1 Patient characteristics in each stage of COVID-19

**Figure 1:**
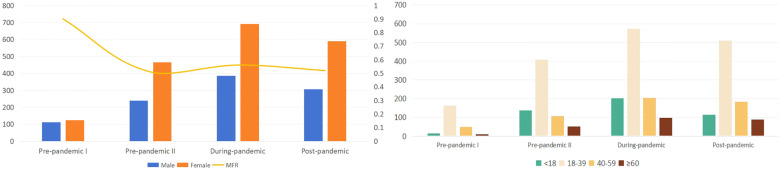
Patient characteristics in each stage of COVID-19.

Between December 1, 2021, and November 30, 2023, 2914 prescriptions were collected; of these, 1042 were for males and 1872 were for females, with a male-to-female ratio (MFR) of 0.56 (**Figure 1**). In pre-pandemic II period, females accounted for the largest proportion (66.10%), and the number of male patients during pandemic period was 385, reaching 35.75%. Young people aged 18-39 accounted for the largest proportion of patients, with a total of 1652 cases (56.70%) (**Table 1**).

**Table 1:** Patient characteristics in each stage of COVID-19.

Type		Pre-pandemic I		Pre-pandemic II		During-pandemic		Post-pandemic		Total	Ratio/%
		cases	Ratio/%	cases	Ratio/%	cases	Ratio/%	cases	Ratio/%		
Gender	Male	112	47.46	239	33.90	385	35.75	306	34.15	1042	35.76
	Female	124	52.54	466	66.10	692	64.25	590	65.85	1872	64.24
	MFR	0.9		0.51		0.56		0.52		0.56	
	Total	236		705		1077		896		2914	
Age	<18	14	5.93	137	19.43	203	18.85	115	12.83	469	16.09
	18-39	162	68.64	409	58.02	572	53.11	509	56.81	1652	56.70
	40-59	50	21.19	108	15.32	205	19.03	184	20.54	547	18.77
	≥60	10	4.24	51	7.23	97	9.01	88	9.82	246	8.44
	Total	236	100	705	100	1077	100	896	100	2914	100

MFR, male-to-female ratio.

### 3.2 Type of prescriptions in each stage of the pandemic

**Figure 2:**
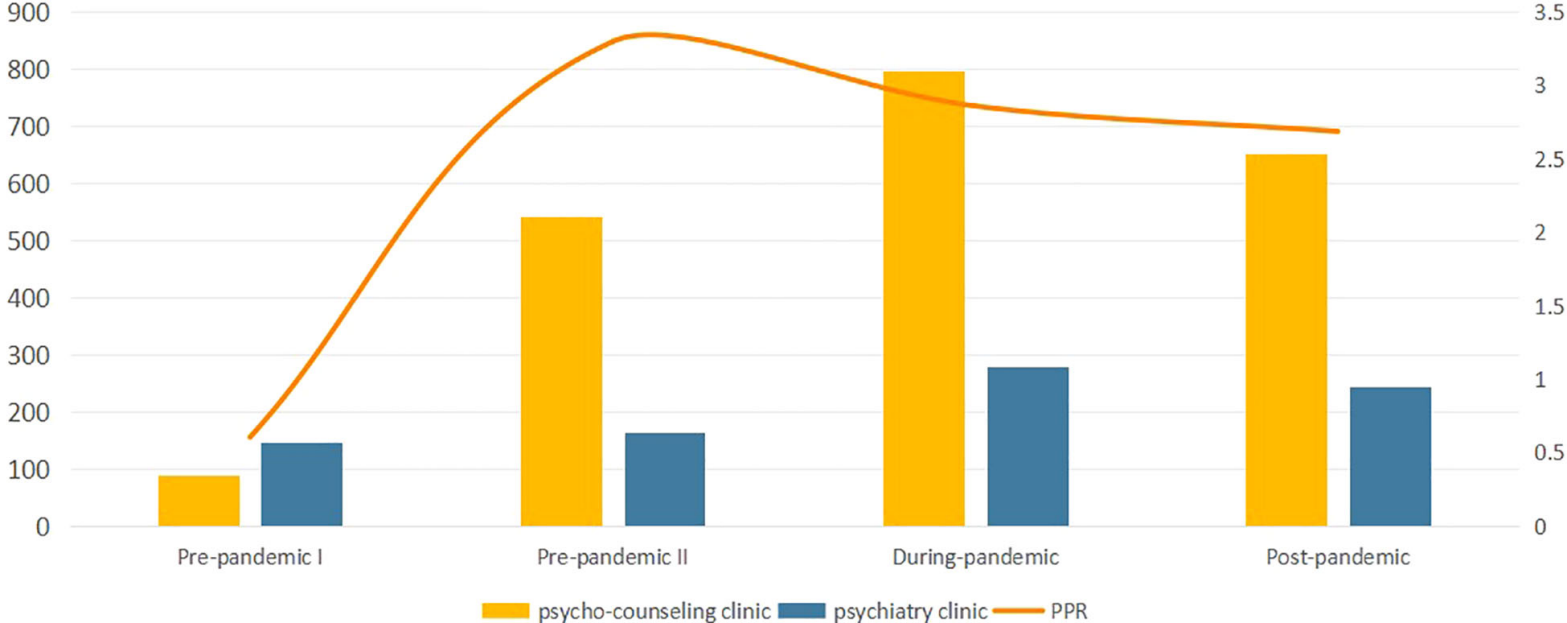
Type of prescriptions in each stage of the pandemic.

The “Internet+” pharmacy in our hospital mainly include two categories: psychiatric clinics and psycho-counseling clinics. Of all the prescriptions, a total of 833 cases were psychiatric prescriptions (28.59%) and 2081 were psycho-counseling prescriptions (71.41%). Psycho-counseling clinic in during-pandemic period accounted the most prescriptions (**Table 2**). In addition, the ratio of psycho-counseling prescription to psychiatric prescription (PPR) in pre-pandemic II was 3.30, indicating the highest proportion of psycho-counseling in this stage (**Figure 2**).

**Table 2:** Type of prescriptions in each stage of the pandemic.

Type	Pre-pandemic I		Pre-pandemic II		During-pandemic		Post-pandemic		Total	Ratio/%
	cases	Ratio/%	cases	Ratio/%	cases	Ratio/%	cases	Ratio/%		
psycho-counseling clinic	89	37.71	541	76.74	798	74.09	653	72.88	2081	71.41
psychiatry clinic	147	62.29	164	23.26	279	25.91	243	27.12	833	28.59
PPR	0.61		3.30		2.86		2.69		2.5	
Total	236	100	705	100	1077	100	896	100	2914	100

PPR, the ratio of psycho-counseling prescription to psychiatric prescription.

### 3.3 Type of diseases in each stage of the pandemic

In 2914 prescriptions, a total of 38 diseases were involved after categorizing the same diseases. Depression, anxiety, schizophrenia were the top three diseases. In during-pandemic period, 433 cases of depression according to symptoms was reported, accounting for 40.20% (**Table 3**).

**Table 3:** Type of diseases in each stage of the pandemic.

Disease type	Pre-pandemic I		Pre-pandemic II		During-pandemic		Post-pandemic		Total	Ratio/%
	case	ratio/%	case	ratio/%	case	ratio/%	case	ratio/%		
Depression	71	30.08	272	38.58	433	40.20	360	40.18	1136	38.98
Anxiety	34	14.41	105	14.89	170	15.78	180	20.09	489	16.78
Schizophrenia	30	12.71	79	11.21	128	11.88	99	11.05	336	11.53
Childhood emotional disorder	5	2.12	49	6.95	59	5.48	42	4.69	155	5.32
Mood affective disorder	7	2.97	32	4.54	69	6.41	41	4.58	149	5.12
Obsessive-compulsive disorder	12	5.09	37	5.26	46	4.27	46	5.13	141	4.84
Bipolar disorder	11	4.66	40	5.67	36	3.34	11	1.23	98	3.36
Tic disorder	0	0	12	1.7	24	2.23	13	1.45	49	1.68
Mental weakness	41	17.37	1	0.14	1	0.10	0	0	43	1.48
Acute and transient psychotic disorder	0	0	6	0.85	15	1.39	15	1.67	36	1.25
Organic mental disorders	1	0.42	10	1.42	12	1.11	10	1.12	33	1.13
Somatoform disorder	3	1.27	4	0.57	15	1.39	8	0.89	30	1.03
Attention deficit and hyperactivity disorder	1	0.42	7	0.99	11	1.03	9	1	28	0.96
Manic episode	6	2.54	10	1.42	2	0.19	8	0.89	26	0.89
Alzheimer’s disease	1	0.43	4	0.57	9	0.84	10	1.13	24	0.83
Sleep disorders	6	2.54	2	0.28	4	0.37	6	0.67	18	0.62
Hysterical psychosis	0	0	2	0.28	5	0.46	7	0.78	14	0.48
Eating disorders	0	0	6	0.85	4	0.38	3	0.33	13	0.45
Mental disorders caused by cerebrovascular disease	0	0	3	0.43	0	0	10	1.12	13	0.45
Mental retardation	0	0	4	0.57	5	0.46	3	0.33	12	0.41
Mental disorders	2	0.86	5	0.71	5	0.46	0	0	12	0.41
Dementia	1	0.42	5	0.71	3	0.28	1	0.11	10	0.34
Panic attack	0	0	0	0	4	0.37	5	0.57	9	0.31
Epileptic psychosis	0	0	1	0.14	3	0.28	3	0.33	7	0.24
Fantasy delusional state	0	0	1	0.14	5	0.46	1	0.11	7	0.24
Asperger’s syndrome	0	0	2	0.28	2	0.19	1	0.11	5	0.17
Paranoid state	1	0.42	3	0.43	0	0	0	0	4	0.14
Childhood autism	0	0	0	0	2	0.19	1	0.11	3	0.1
Hysteria	0	0	0	0	2	0.19	0	0	2	0.07
Cognitive impairment	0	0	1	0.14	1	0.09	0	0	2	0.07
Hypochondriasis	2	0.85	0	0	0	0	0	0	2	0.07
Delirium	0	0	0	0	0	0	2	0.22	2	0.07
Separate convulsions	0	0	1	0.14	0	0	0	0	1	0.03
Hypertension	0	0	0	0	1	0.09	0	0	1	0.03
Secondary insomnia	0	0	0	0	1	0.09	0	0	1	0.03
Neurogenic headache	0	0	1	0.14	0	0	0	0	1	0.03
Eczema	1	0.42	0	0	0	0	0	0	1	0.03
Adaptation disorder	0	0	0	0	0	0	1	0.11	1	0.03
Total	236	100	705	100	1077	100	896	100	2914	100

### 3.4 Medication types in each stage

A total of 4404 drugs were prescribed. The medication type mainly concentrated in antidepressant (49.60%), antipsychotics (34.13%) and antiepileptic drugs (4.70%). The proportion of antidepressant was the highest in each stage, and 726 cases of antidepressant were prescribed in post-pandemic period, accounted for 54.59% (**Table 4**).

**Table 4:** Medication types in each stage.

Types	Pre-pandemic I		Pre-pandemic II		During-pandemic		Post-pandemic		Total	Ratio/%
	case	ratio/%	case	ratio/%	case	ratio/%	case	ratio/%		
Antidepressant	142	43.56	493	44.86	824	49.97	726	54.59	2185	49.60
Antipsychotics	128	39.26	397	36.12	563	34.14	415	31.2	1503	34.13
Antiepileptic drugs	14	4.29	71	6.46	68	4.12	54	4.06	207	4.70
Anxiolytics	5	1.53	20	1.82	43	2.61	50	3.76	118	2.68
Antimanic drugs	8	2.45	33	3	36	2.18	25	1.88	102	2.31
Sedative drugs	5	1.53	30	2.73	29	1.76	12	0.9	76	1.73
Antidementia drugs	2	0.61	9	0.82	10	0.61	10	0.75	31	0.7
Anti-ADHD drugs	1	0.31	7	0.64	9	0.55	10	0.75	27	0.61
Anticholinergic drugs	0	0	5	0.45	11	0.67	11	0.83	27	0.61
Antidepressant Chinese drug	3	0.92	4	0.36	14	0.85	4	0.3	25	0.57
Antiplatelet drugs	1	0.31	10	0.91	7	0.42	7	0.52	25	0.57
Antihypertensive drugs	1	0.31	2	0.18	16	0.97	0	0	19	0.43
Brain nutrition drugs	3	0.93	2	0.18	4	0.24	3	0.23	12	0.27
Improve cerebrovascular drugs	0	0	7	0.65	3	0.18	0	0	10	0.23
Laxatives	7	2.15	0	0	0	0	0	0	7	0.16
Antidiabetic drug	0	0	4	0.37	2	0.12	0	0	6	0.14
Blood system drugs	0	0	0	0	6	0.37	0	0	6	0.14
Hepatic protector	0	0	3	0.27	1	0.06	2	0.15	6	0.14
Vitamins	2	0.61	1	0.09	0	0	0	0	3	0.07
Antibacterial drug	2	0.61	0	0	0	0	0	0	2	0.05
Heart-protecting drugs	1	0.31	0	0	1	0.06	0	0	2	0.05
Hormone drugs	0	0	0	0	1	0.06	1	0.08	2	0.05
Anti-cold drugs	1	0.31	0	0	0	0	0	0	1	0.02
Blood circulation drugs	0	0	1	0.09	0	0	0	0	1	0.02
Cardiovascular drug	0	0	0	0	1	0.06	0	0	1	0.02
Total	326	100	1099	100	1649	100	1330	100	4404	100

ADHD, Attention-Deficit/Hyperactivity Disorder.

### 3.5 Medication use in each stage

Among the 4404 prescriptions, 85 kinds of medication were involved. The top 10 medications were shown in **Table 5**, with quetiapine, aripiprazole, and escitalopram represent as the highest top three frequency medications. In post-pandemic period, escitalopram accounted for the majority (10.98%), with 146 cases.

**Table 5:** Distribution of the top 10 medications in each stage of COVID-19.

Medication	Pre-pandemic I		Pre-pandemic II		During-pandemic		Post-pandemic		Total	Ratio/%
	case	ratio/%	case	ratio/%	case	ratio/%	case	ratio/%		
Quetiapine	54	16.56	120	10.92	148	8.98	124	9.32	446	10.13
Aripiprazole	29	8.9	105	9.55	164	9.95	124	9.32	422	9.58
Escitalopram	33	10.12	86	7.83	142	8.61	146	10.98	407	9.24
Duloxetine	23	7.06	73	6.64	154	9.34	117	8.8	367	8.33
Olanzapine	28	8.59	96	8.74	114	6.91	70	5.26	308	6.99
Venlafaxine	18	5.52	73	6.64	73	4.43	97	7.29	261	5.93
Fluvoxamine	14	4.29	42	3.82	84	5.09	72	5.41	212	4.81
Agomelatine	9	2.76	34	3.09	90	5.46	76	5.71	209	4.75
Sertraline	11	3.37	49	4.46	67	4.06	68	5.11	195	4.43
Mirtazapine	14	4.29	52	4.73	51	3.09	29	2.18	146	3.32

### 3.6 Medication frequency in each stage

The frequency of patients taking drugs in each stage of the epidemic was shown in **Table 6**. Among them, taking medicine once a day was the most common frequency, accounting for 70.87%.

**Table 6:** Medication frequency in each stage.

Frequency	Pre-pandemic I		Pre-pandemic II		During-pandemic		Post-pandemic		Total	Ratio/%
	cases	Ratio/%	cases	Ratio/%	cases	Ratio/%	cases	Ratio/%		
Once a day	238	73.01	767	69.79	1156	70.1	960	72.18	3121	70.87
Twice a day	67	20.55	255	23.3	375	22.74	276	20.75	973	22.09
Three times a day	20	6.13	74	6.73	117	7.1	92	6.92	303	6.88
Prorenata	0	0	3	0.27	0	0	0	0	3	0.07
Once every other day	0	0	0	0	1	0.06	0	0	1	0.02
Interval of one week	0	0	0	0	0	0	2	0.15	2	0.05
Twice a week	1	0.31	0	0	0	0	0	0	1	0.02
Total	326	100	1099	100	1649	100	1330	100	4404	100

### 3.7 Pharmacist review time in each stage

Pharmacist review time in each stage was shown in **Table 7**. 42.23% of the prescription review time was less than or equal to one minute, and 31.18% of the prescriptions was 1-10 minutes. It is worth noting that 14.42% of the prescription review time was more than 60 minutes. The mean, median and IQR of pharmacist review time in post-pandemic period was the highest, representing as 6175.1 s, 305.5 s, and 4148.5 s, respectively (**Table 7**). 12:00-17:59 was the most (41.46%) pharmacist review time point, with 1147 cases, while in post-pandemic period, 320 prescriptions were reviewed between 18:00-23:59, accounting for 36.70% (**Table 8**).

**Table 7:** Pharmacist review time in each stage.

Time	Pre-pandemic I		Pre-pandemic II		During-pandemic		Post-pandemic		Total	Ratio
	case	ratio/%	case	ratio/%	case	ratio/%	case	ratio/%		
≤1min	102	50.25	427	62.79	446	43.22	202	23.17	1177	42.23
1-10min	76	37.44	138	20.29	372	36.05	283	32.45	869	31.18
10-30min	12	5.91	33	4.85	71	6.88	85	9.75	201	7.21
30-60min	3	1.48	27	3.97	35	3.39	73	8.37	138	4.95
>60min	10	4.93	55	8.09	108	10.47	229	26.26	402	14.42
Mean (s)	1371.6		1535.5		2587.1		6175.1			
Median (s)	59		44		72		305.5			
IQR (s)	121.5		168		220.5		4148.5			
Total	203	100	680	100	1032	100	872	100	2787	100

IQR, interquartile range.

There were 127 prescriptions not included because they did not support to review.

**Table 8:** Pharmacist review time point in each stage.

Time	Pre-pandemic I		Pre-pandemic II		During-pandemic		Post-pandemic		Total	Ratio
	case	ratio/%	case	ratio/%	case	ratio/%	case	ratio/%		
00:00-05:59	1	0.49	3	0.44	12	1.16	13	1.49	29	1.04
06:00-11:59	54	26.6	163	23.97	245	23.74	226	25.92	688	24.69
12:00-17:59	90	44.34	311	45.74	433	41.96	313	35.89	1147	41.16
18:00-23:59	58	28.57	203	29.85	342	33.14	320	36.70	923	33.12
Total	203	100	680	100	1032	100	872	100	2787	100

There were 127 prescriptions not included because they did not support to review.

### 3.8 Types of irrational medication use

The issue of irrational medication use included OLI, MESP, DDI, and UDE. Of all the 2914 prescriptions, 1103 (37.85%) were not initially approved by the system; 68.63% of irrational prescriptions represent as one irrational problem. The majority of irrational prescriptions was OLI, accounting for 50.86%, and followed by MESP and OLI, which accounted for 16.60% (**Table 9**). After re-examination by pharmacists, the number of irrational prescriptions decreased to 33, accounting for 1.13% of the total prescriptions (**Table 10**). UDE, OLI and DDI were the main irrational types.

**Table 9:** Types of irrational medication use reviewed by system.

	Item	Pre-pandemic I		Pre-pandemic II		During-pandemic		Post-pandemic		Total	Ratio/%
		case	ratio/%	case	ratio/%	case	ratio/%	case	ratio/%		
Type	OLI	58	70.72	118	43.7	236	52.56	149	49.34	561	50.86
	OLI; MESP	6	7.32	66	24.44	56	12.47	55	18.22	183	16.60
	MESP	7	8.54	28	10.38	65	14.48	34	11.26	134	12.15
	OLI; DDI	1	1.22	28	10.38	41	9.13	38	12.58	108	9.79
	DDI	2	2.44	12	4.44	21	4.68	22	7.28	57	5.17
	MESP; DDI	0	0	8	2.96	12	2.67	3	0.99	23	2.09
	OLI; MESP; DDI	0	0	9	3.33	11	2.45	1	0.33	21	1.9
	OLI; UDE	7	8.54	0	0	0	0	0	0	7	0.63
	UDE	0	0	1	0.37	4	0.89	0	0	5	0.45
	OLI; UDE; DDI	0	0	0	0	2	0.45	0	0	2	0.18
	MESP; UDE	1	1.22	0	0	1	0.22	0	0	2	0.18
	Total	82	100	270	100	449	100	302	100	1103	100
Number of irrational problems	1	67	81.71	159	58.89	326	72.60	205	67.88	757	68.63
	2	15	18.29	102	37.78	110	24.50	96	31.79	323	29.28
	3	0	0	9	3.33	13	2.90	1	0.33	23	2.09
	Total	82	100	270	100	449	100	302	100	1103	100

OLI, off-label indications.

MESP, medication errors of special population.

DDI, drug-drug interaction.

UDE, usage and dosage errors.

**Table 10:** Types of irrational medication use reviewed by pharmacist.

Types	Pre-pandemic I		Pre-pandemic II		During-pandemic		Post-pandemic		Total	Ratio/%
	case	ratio/%	case	ratio/%	case	ratio/%	case	ratio/%		
UDE	2	100	2	25	11	57.89	2	50	17	51.52
OLI	0	0	4	50	8	42.11	1	25	13	39.39
DDI	0	0	2	25	0	0	0	0	2	6.06
MESP	0	0	0	0	0	0	1	25	1	3.03
Total	2	100	8	100	19	100	4	100	33	100

OLI, off-label indications.

MESP, medication errors of special population.

DDI, drug-drug interaction.

UDE, usage and dosage errors.

## 4 Discussion

How to minimize cross-infection posed a significant challenge for governments worldwide during the COVID-19 pandemic. “Internet+” medical care emerged as a feasible solution, bridging the gap between preventing the spread of the virus and ensuring access to medical treatment (13). To reduce the risk of cross-infection within hospitals and curb virus transmission through interpersonal contact, medical institutions in China implemented remote pharmacy services during the pandemic, including online consultations, prescribing, and drug delivery services (14, 15). However, in the post-pandemic era, it remains unclear whether the rapid growth of “Internet+” medical care can continue to meet patients’ needs. Most existing studies focus on the introduction of Internet medical models (16), their impact on offline medical services (17), and their operational status (18), with little attention paid to the analysis of Internet prescriptions in psychiatry during the post-pandemic era. This study addresses this gap by conducting a comprehensive analysis of psychiatric Internet prescriptions, categorized by different periods, and examining variables such as patient gender, age, prescription type, consultation type, medication categories, pharmacist review time, and types of irrational prescriptions. Evaluating the changes in Internet prescriptions before and after the pandemic can provide valuable insights for the future development of “Internet+” pharmacy.

Our study identified differences in the MFR and prescriptions across four distinct stages. During the pre-pandemic I period, the number of Internet prescriptions for males and females was almost identical, with an MFR of 0.90. However, the number of prescriptions increased significantly during the pre-pandemic II period, with the rise being predominantly attributed to women, causing the MFR to decrease to 0.51. Given the ongoing impact of COVID-19 and strict social isolation measures, public mental health was severely affected (19). Notably, in December 2022, China relaxed its COVID-19 control measures, which may have significantly increased public panic and risk perception, contributing to a surge in psychological issues during the pre-pandemic II period (20). Research has shown that women are more vulnerable to external environmental stressors than men, leading to emotional fluctuations and mental health issues (21), such as anxiety and depression (22). Furthermore, the increased caregiving burden on women has exacerbated their psychological stress (23). Help-seeking behavior is another important aspect. Studies have indicated that women were more likely to seek help during the COVID-19 pandemic, and the use of technology-driven solutions in “Internet+” healthcare targeting women’s health accelerated (24), which aligns with our findings. During the epidemic period, the MFR increased slightly from 0.51 to 0.56, with a significant rise in both male and female patients. The slight increase in the proportion of male patients suggests that men may have faced greater psychological pressure during the pandemic. This may be due to the disruption of work and life caused by epidemic control measures, leading to social issues such as higher male unemployment rates (25). Consequently, the survival pressures on men increased, contributing to a rise in psychological problems.

In addition to the changes in MFR, we found that individuals aged 18-39 made up the majority of patients. This age group faced significant risks during the epidemic, including unemployment and academic disruptions. Over the past decade, depression rates among young people have risen sharply (26), leading to adverse outcomes that persistently affect interpersonal relationships, education, and occupational functioning. Fortunately, compared to older adults, young people are more likely to utilize “Internet+” hospitals (27). Therefore, prevention and early intervention in “Internet+” medical care for this age group should be strengthened, with a focus on patients with a family history of depression and those exposed to negative life events. More importantly, proactive universal prevention strategies should be implemented. Online psycho-counseling can serve as an initial intervention in a stepwise treatment approach (28).

The number of psycho-counseling prescriptions in the pre-pandemic II period increased compared to the pre-pandemic I period. Additionally, the PPR was highest during pre-pandemic II, followed by a slight decrease during the epidemic and post-epidemic periods. This could be attributed to the high level of public trust in the Chinese political system, which, combined with the perceived risk of COVID-19, may have led to an increase in over-preventive behavior (29). However, the effectiveness of this over-prevention remained uncertain before the epidemic measures were lifted. This uncertainty—particularly the unpredictability and lack of control over contracting the virus—could have contributed to increased negative emotions (30), leading to a rise in psycho-counseling prescriptions during pre-pandemic II. The increase in PPF resulted from the relatively stable psychiatric prescriptions and the rising number of psycho-counseling prescriptions, which further underscored the mental health impact of COVID-19. The number of psycho-counseling prescriptions during the epidemic increased compared to pre-pandemic II but declined in the post-pandemic period. A review of mental health in affluent European nations indicated that the prevalence of certain mental health issues was higher during the pandemic than before, but this increase generally subsided over time (4). This finding aligns with our research. During the pandemic period, although the number of psycho-counseling prescriptions continued to rise, the overall number of psychiatric patients increased. This led to a reduction in the PPR, as the total number of patients in the “Internet+” hospital grew.

In different stage of the epidemic, the top three diseases were depression, anxiety and schizophrenia. COVID-19, as a major public health emergency of this century, has had a significant impact on mental health. Public health emergencies can lead to an increase in mental health issues (31) and a decline in economic conditions (32). Additionally, measures such as social distancing and isolation are risk factors for mental disorders, contributing to feelings of loneliness, reduced social support, and insufficient detection of mental health needs (33). Regarding medication categories, antidepressants and antipsychotics were the two most commonly prescribed types of drugs. Among these, quetiapine, aripiprazole, and escitalopram were the most frequently used antipsychotics and antidepressants. Most of the top ten drugs prescribed were antidepressants, antipsychotics, and anxiolytics, which align closely with the classification of the diseases. This reflects the mental health challenges posed by COVID-19 and the treatment needs of patients in “Internet+” hospitals. A once-daily medication regimen was the most common prescription frequency across all stages, which is conducive to improving patient medication adherence.

As a critical component of prescription circulation, prescription review plays an irreplaceable role in ensuring the safety and effectiveness of patients’ medications. The prescription review system in our hospital evaluates the rationality of prescriptions before they are reviewed by pharmacists. The initial rational prescription rate identified by the system was 62.15%. After manual review by pharmacists, the final rational prescription rate increased to 98.87%. This significant difference in qualification rates highlights the limitations of the system review. The system review primarily relies on predefined algorithms and data rules to automatically detect potential medication issues (e.g., drug interactions, inappropriate dosages). However, the accuracy of these judgments is constrained by the design of the algorithms and the quality of the data. In contrast, pharmacist reviews incorporate clinical experience and consider individual patient circumstances, offering greater flexibility and professional judgment. When faced with complex or special cases, pharmacists may make decisions that differ from those of the system, which contributes to discrepancies in rationality rates. Our study suggests that the strict standards of the system review may lead to some rational prescriptions being flagged as irrational, while pharmacists are able to adjust their judgments based on specific circumstances. Therefore, while the prescription review system offers an important tool, its accuracy needs further improvement, and pharmacists remain essential in ensuring the quality of prescription reviews.

OLI was the most common form of irrational medication use in the systematic prescription review, which may be related to the untimely update of the prescription review database (34). After pharmacist review, UDE became the most common irrational prescription. To address irrational prescriptions, clinical prescription management and review processes should be strengthened, and irrational prescriptions should be returned promptly to ensure accuracy. In addition, pharmacists should actively maintain the prescription review system to bridge the differences between clinical practice and the database. The review logic of the system can be improved by incorporating more clinical variables and individual patient characteristics, enabling it to better evaluate complex cases. If necessary, pharmacists can facilitate internal hospital meetings to gather feedback from doctors. Furthermore, artificial intelligence could be utilized to help develop a more robust Internet prescription review platform (11).

During the pharmacists’ prescription review process, the number of prescriptions with a review time exceeding 60 minutes increased during the epidemic. This prolonged review time may have been due to the surge in pharmacists’ workloads and personnel shortages during the pandemic. The increase in complex prescriptions resulting from COVID-19 could also have contributed to the extended review times. It is important to note that this issue persisted in the post-pandemic period. This contradicts our typical experience, as review times usually decrease with increased years of work and experience. Moreover, we found that the mean, median, and IQR of review times were highest in the post-pandemic period, suggesting a large number of prescriptions exceeded the 60-minute threshold. This finding highlights the growing workload of pharmacists. Additionally, the most common time for pharmacists to review prescriptions shifted from 12:00-17:59 during the epidemic to 18:00-23:59 in the post-pandemic period, indicating that pharmacists were reviewing more prescriptions after regular working hours. Previous studies have shown that pharmacists were at risk of burnout even before the pandemic (35). The increased workload caused by the epidemic, coupled with reduced rest time, has likely aggravated job burnout (36), which may have impacted the overall prescription review time.

In post-pandemic period, although the number of prescriptions was fewer than that in during-pandemic period, it still represented a significant rise compared with that before the pandemic, indicating a high demand and acceptance of “Internet+” medical care after COVID-19. The number of female patients rose significantly compared to males, with a greater increase in women compared to the pre-pandemic I period. Young people aged 18-39 remained the primary patient group in “Internet+” hospitals. Additionally, psycho-counseling prescriptions outnumbered psychiatric prescriptions, and psycho-counseling prescriptions saw a significant increase compared to before the epidemic. Looking ahead, the allocation of Internet medical resources should involve increasing relevant departments and personnel to meet the growing demand. Moreover, the number of patients diagnosed with depression and receiving antidepressants has risen compared to pre-epidemic levels, with escitalopram use increasing notably. Pharmacists should adjust the “Internet+” hospital medication catalog to meet clinical needs. Given the increased use of drug combinations and the risk of serotonin-related side effects, clinical pharmacy training on escitalopram and related antidepressants should be enhanced to mitigate adverse reactions such as serotonin syndrome (37). Administering medications once a day aligns with patient medication habits, improving adherence and reducing the risk of adverse effects. It is also worth noting that prescription review time in “Internet+” pharmacies increased significantly compared to the pre-pandemic period. To address this, measures such as increasing the number of pharmacists, refining rules and regulations, optimizing workflow, offering adequate vacation time, ensuring fair salaries (36), and fostering a sense of professional pride among pharmacists can help reduce job burnout and shorten review times.

The COVID-19 pandemic has accelerated the development of “Internet+” healthcare (38, 39) and profoundly altered the medical landscape of “Internet+” hospitals. These hospitals have balanced epidemic prevention with patient care by reducing the risk of patient aggregation and cross-infection (13). According to a study by the International Pharmaceutical Federation, “Internet+” pharmaceutical care is expected to become a key component of telemedicine in the coming years. It can provide patients with more timely drug care, lower individual and healthcare system costs, improve patient satisfaction and convenience, and lead to better health outcomes (40). Additionally, numerous studies have confirmed that “Internet+” pharmaceutical care addresses pharmacist shortages and ensures appropriate drug assistance in underserved areas (41, 42). The rise of “Internet+” pharmaceutical care has accelerated the transformation of pharmacists’ roles (43). Overall, “Internet+” hospitals have significantly improved the medical experience for patients during the epidemic and played a crucial role in COVID-19 prevention and control (16).

Although this study revealed differences in Internet prescriptions in psychiatric hospitals before and after the pandemic, its single-center design limits external validity due to the lack of validation from different regions or hospitals. To address this, future research will adopt a multi-center design. We have already submitted an ethics application to the hospital and plan to collect broader data through collaborating hospitals. Additionally, the current study did not incorporate quantitative analysis, which limits the depth and comprehensiveness of the explanations for the observed phenomena. Therefore, subsequent research will employ a mixed-methods approach, integrating quantitative data with qualitative interviews to further explore and elucidate the underlying causes of these phenomena, thereby enhancing the depth and comprehensiveness of the findings.

## 5 Conclusion

The integration of medical health services with the Internet is pivotal in achieving convenient, precise, and intelligent healthcare services, aligning with the goals of “Healthy China 2030.” This large-scale adoption of “Internet+” healthcare during a unique period demonstrates the collaborative efforts of China’s medical system and the public, further driving the advancement of “Internet+” healthcare services. Among these, “Internet+” pharmacy has played a significant role through its synergy with online diagnosis and treatment. It has not only expanded communication channels between clinicians and patients but also effectively reduced the pressure of patients congregating for in-person medical care, while addressing their healthcare needs.

This study analyzed the prescriptions and pharmaceutical services of Internet-based psychiatric hospitals in relation to the COVID-19 development timeline, including prescription volume, drug types, prescription reviews, and pharmacists’ review time. Specifically, it examined the differences in prescriptions before, during, and after the pandemic, addressing a gap in this field. Moving forward, “Internet+” pharmacy initiatives can focus on improving staffing levels and actively expanding services to include online pharmaceutical clinics and pharmaceutical care, paving the way for a novel pharmaceutical care model in the “Internet+” pharmacy ecosystem.

## Data Availability

The original contributions presented in the study are included in the article/Supplementary Material. Further inquiries can be directed to the corresponding authors.
